# Associations among familism, maternal parenting, and weight-related behaviors in Mexican American adolescents: a cross-sectional structural equation modeling study

**DOI:** 10.1186/s12889-026-27009-9

**Published:** 2026-03-25

**Authors:** Kay W. Kim, Deborah Wiebe, Katherine R. Kogut, Kim G. Harley, Nina T. Holland, Brenda Eskenazi, Julianna Deardorff

**Affiliations:** 1 Department of Psychological Sciences, University of California, Merced, 5200 North Lake Road, Merced, CA 95343 USA; 2 School of Public Health, University of California, Berkeley, 2121 Berkeley Way, Berkeley, CA 94720 USA

**Keywords:** Familism, Communication, Monitoring, Adolescent, Diet, Physical activity, Screen time, Sleep, Weight, Obesity prevention

## Abstract

**Background:**

Mexican American adolescents in the U.S. face disproportionately high rates of obesity. Familism, a core Mexican cultural value emphasizing family closeness, and parenting processes such as communication and monitoring are associated with adolescents’ weight-related behaviors such as diet, physical activity, sleep, screen time and weight outcomes.

**Methods:**

Participants were 439 mother-adolescent dyads (47.2% boys) from the CHAMACOS cohort, a longitudinal study of Mexican-origin families in California’s Salinas Valley. Data were collected at the 14-year study visit. Adolescents reported on familism, maternal communication and monitoring, and their own weight-related behaviors. Anthropometric data were collected to calculate BMI percentiles. Structural equation modeling was used to test hypothesized relationships among variables and examine gender differences.

**Results:**

Familism was positively associated with maternal communication and monitoring, as well as with adolescent physical activity and sleep, and negatively with screen time. Mother-youth communication was related to longer sleep duration, while maternal monitoring was not significantly related to any weight-related behaviors. Fruit and vegetable intake and sleep were negatively associated with weight status. Gender differences were observed: for girls, mother-daughter communication was linked to longer sleep duration, and for boys, familism was related to higher physical activity.

**Conclusions:**

The results highlight significant associations between cultural values, maternal parenting, and healthy weight-related behaviors among Latino adolescents. These findings emphasize that incorporating gender-sensitive cultural and family dynamics may enhance the design of obesity prevention strategies for this population.

Mexican American adolescents face a disproportionately high burden of obesity, with a prevalence of 25.8% compared to 18.5% in the general population, along with significant increases in severe obesity over the past decades [1]. This heightened prevalence of obesity contributes to the increased risk of hypertension, dyslipidemia, and impaired glucose tolerance among this population [2, 3]. Beyond physical health, Latino adolescents with obesity encounter substantial psychological challenges, such as reduced quality of life and social isolation, largely stemming from pervasive societal weight-stigma and discrimination [4–6]. Importantly, obesity during adolescence is a strong predictor of obesity in adulthood [7]. Despite the risk, many adolescents engage in unhealthy weight-related behaviors during this developmental period, such as insufficient consumption of fruits and vegetables, and frequent consumption of fast food. Adolescents also tend to have reduced physical activity, which may be exacerbated by reduced sleep and excessive screen time [8]. These trends underscore the urgent need to understand mechanisms of Latino adolescent obesity and weight-related behaviors to develop effective health promotion strategies.

Understanding the health and well-being of racial and ethnic minority groups through the lens of culture is vital for promoting cultural competence in healthcare delivery and fostering meaningful community engagement. Culture encompasses the shared beliefs, values, customs, norms and practices that characterize a particular group of people. It shapes individuals’ behaviors, attitudes and interactions within their social environments [9–11]. These cultural influences can affect how adolescents communicate with parents, seek support, and interact with healthcare providers [12, 13]. Given culture’s central role in health behaviors [12], there have been calls to incorporate cultural understanding into health studies [14–16]. However, empirical research integrating these cultural nuances remains scarce.

Familism is a core value among Latino communities and is prominent among Mexican American families in the U.S [17, 18]. Familism is defined as a value system that emphasizes strong attachment to and identification with the family, promoting warm, close, and supportive relationships that prioritize family over the self [14, 19]. People with high levels of familism endorse family interconnectedness, use the family unit as a key personal referent and respect and uphold family honor [19, 20]. As familism emphasizes family cohesion and collective identity, it encourages and promotes healthier behaviors, such as healthy dietary intake and exercise [21] and monitoring of health practices [22], by strengthening family support and promoting prosocial, health-oriented routines. Further research is warranted to examine how familism is related to adolescents’ weight-related behaviors and weight status.

Furthermore, parenting processes, which are shaped by cultural values such as familism, have a substantial influence on adolescent weight-related behaviors, including food intake, physical activity, screen time, sleep and overall weight status [23, 24]. Specifically, two parenting factors have been identified to have compelling links to adolescent weight-related behaviors: parental monitoring and parent-adolescent communication [25–27]. Parental monitoring encompasses parents’ proactive involvement in understanding their adolescents’ whereabouts and activities during their free time achieved through direct supervision and communication with the adolescent [28]. This active parental awareness is crucial for adolescents’ daily behaviors, including those related to weight [25–27]. For instance, parental monitoring was associated with greater consumption of healthy foods and higher levels of physical activity, as well as reduced intake of unhealthy foods and lower screen time among Latino adolescents [27].

Another important contextual factor associated with adolescents’ weight-related behaviors is parent-adolescent communication. Even when conversations are not explicitly about health, the quality of everyday communication establishes family norms and routines that can promote or hinder these behaviors. For instance, prior studies have demonstrated that effective parent–adolescent communication is associated with healthier weight-related behaviors, such as decreased fast food intake and increased engagement in physical activity [26]. Also, more communication with parents was related to longer sleep duration among Latinx adolescents [29] and decreased screen time [30]. As such, parental monitoring and parent-youth communication should be examined as key aspects of the home environment that may lay the foundation for adolescents’ engagement in healthy weight-related behaviors. Specifically, we focused on maternal parenting behaviors as mothers are the primary caregiver responsible for value transmission, socialization of health behaviors and most aspects of family daily in Mexican American households [31, 32].

Moreover, it is crucial to investigate how the relationships among familism, mother-adolescent communication, maternal monitoring and weight-related behaviors may differ between boys and girls. Gender schema theory suggests that individuals are socialized from an early age to adopt specific gender norms, which encompasses socially constructed roles and stereotypes associated with gender [33]. In many Latino cultures, gender norms strongly influence weight-related behaviors. For example, consuming meat, fast food and sugar-sweetened beverages is often socially associated with masculinity, whereas eating vegetables, fruits and other healthy foods is socially perceived as more feminine [34]. Similarly, vigorous team sports are traditionally seen as masculine, which may discourage girls from participating in these activities [35, 36]. Given these deeply ingrained social norms, boys and girls often exhibit distinct patterns of weight-related behaviors and risk. For instance, Latino boys tend to show higher obesity prevalence, whereas girls more frequently report body dissatisfaction and disordered eating behaviors. Failing to account for these gendered expectations could obscure important differences in how parenting factors are related to adolescent weight status.

Along with cultural values encompassing familism, the present study is also informed by broader ecological and relational frameworks. For instance, Bronfenbrenner’s Ecological Theory provides a basis for examining the interplay between individual and familial factors, such as parenting practices, in relation to adolescent behaviors [37, 38]. Similarly, Family Systems Theory emphasizes cohesion and interdependence, suggesting that maternal communication and monitoring are embedded within a broader relational system [39]. Guided by these frameworks, we proposed the conceptual model depicted in Fig. 1. Because the present data are cross-sectional, the study sought to examine associations among familism, maternal parenting processes, adolescent weight-related behaviors and weight-status. This model leads to four specific hypotheses:

- (H1) Familism is positively associated with mother-adolescent communication and maternal monitoring.
- (H2) Familism and maternal parenting processes are positively associated with health-promoting behaviors such as fruit and vegetable intake, physical activity and sleep.
- (H3) Familism and maternal parenting processes are inversely related to health-harming behaviors such as fast-food intake and screen time.
- (H4) Health-promoting behaviors are negatively and health-harming behaviors are positively related to adolescent weight status.
- (H5) The hypothesized pathways between familism, parenting processes, and weight-related behaviors will differ between boys and girls.

**Fig. 1:**
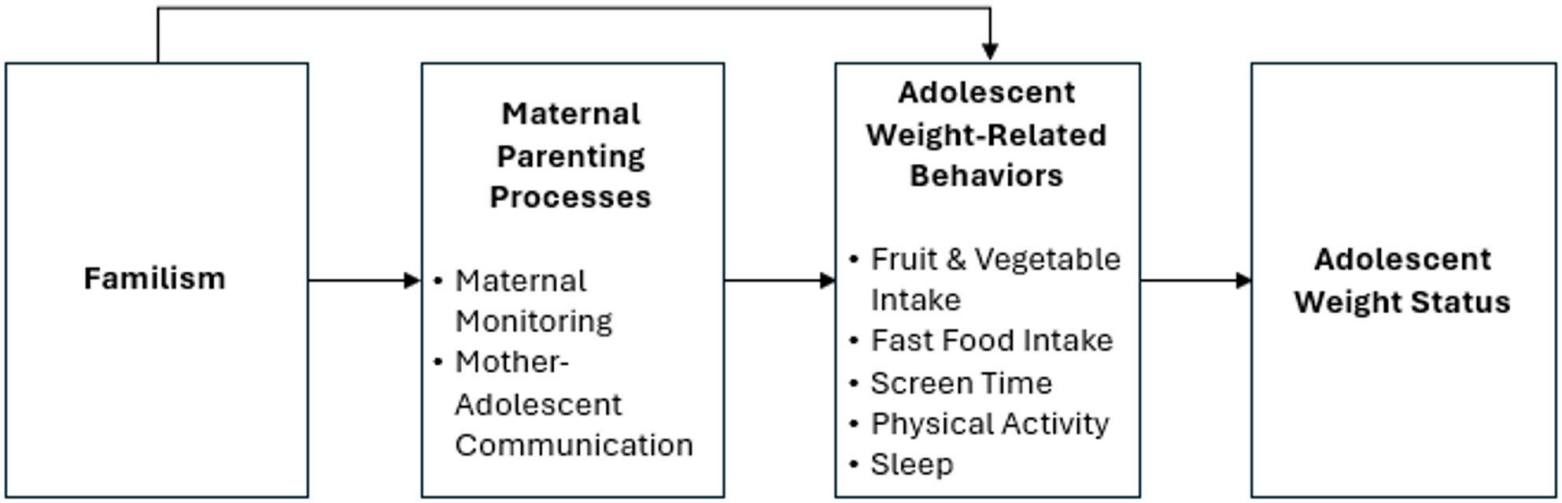
Conceptual model identifying hypothesized relationships

## Materials and methods

### Sample recruitment and data collection procedures

The data were collected through the Center for the Health Assessment of Mothers and Children of Salinas (CHAMACOS) Study, a longitudinal birth cohort study conducted by researchers at the University of California, Berkeley, School of Public Health and Clinica de Salud del Valle de Salinas. The founding purpose of CHAMACOS was to assess the impact of early life pesticide exposures on children’s health and development. Recruitment of mother-child dyads and data collection took place in the Salinas Valley, an agricultural region in Monterey County, California. All CHAMACOS children were born in years 2000–2002, though they were recruited in two phases. A prenatal recruitment effort was conducted in 1999–2000, during which women obtaining prenatal care at local clinics were invited to participate if they were at least 18 years old, less than 20 weeks pregnant, met low-income requirements for state-subsidized medical insurance, spoke either Spanish or English, and planned to deliver at the local safety net hospital. Of 1130 women who met eligibility criteria, 601 enrolled, 532 were followed to delivery of a liveborn singleton or twins (537 infants total with 5 twin pairs), and 353 children were still active in the study at or after age 9 years. The existing cohort was enlarged between 2009 and 2011 through the recruitment of demographically similar families with 9-year-old children. Mother-child dyads were recruited from elementary schools, community events, and through word-of-mouth and were eligible to participate if mothers had received prenatal care in the Salinas Valley, had been 18 or older at delivery, had qualified for state-subsidized medical insurance when pregnant, and spoke Spanish or English. Ultimately, 305 new children were recruited at this age point. At and after child age 9, all mother-child dyads completed the same data collection activities, which were conducted on an approximately annual or biennial basis.

The data used in this analysis were collected between 2014 and 2016 when adolescents were 14 years old. The 14-year study visits included anthropometric measurements of both mothers and youth conducted by trained research staff. A maternal demographic questionnaire (used in the current study as covariates such as family income and education level) was administered in the mother’s preferred language (Spanish or English) by a bilingual, bicultural interviewer, and a youth questionnaire was administered in English, the language of instruction at school. All mothers provided informed consent and parent permission; youth provided written assent. All study procedures were approved by the UC Berkeley Office for the Protection of Human Subjects. The study was preregistered [40].

### Study measures

#### Mexican cultural values scale

At the study visit, youth responded to a shortened version of the Mexican Cultural Values Scale (MCVS; [41]). The MCVS was developed by researchers in Arizona based on focus groups with Mexican American parents and adolescents regarding their perceptions of key Mexican values. CHAMACOS participants completed three subscales assessing different aspects of *familismo*: Familism-Obligation (5 items; e.g., “If a relative is having a hard time financially, one should help them out if possible”), Familism-Referents (5 items; e.g., “A person should always think about their family when making important decisions”), and Familism-Support (6 items; e.g., “Parents should teach their children that the family always comes first”). For mothers, the items were typically administered aloud by study interviewers, and participants responded according to a 5-point Likert scale, ranging from 1 (not at all) to 5 (very much). The scale demonstrated good internal consistency, with a Cronbach’s alpha of 0.80.

#### Maternal monitoring

At the study visit, youth completed the Small Parental Monitoring Scale [42] was administered by a study interviewer, with youth providing reflections on their mothers’ monitoring behaviors. The scale included 10 items rated on a 5-point Likert scale (e.g., “Your mother knew what you were doing after school,” “Your mother shows an interest in who you spend your time with, “You and your mother discuss your social plans”; Almost never or never, Once in a while, Sometimes, A lot of time, Almost always or always). Scores range from 1 to 5, with 5 reflecting the highest degree of monitoring. Summary scores were computed as an average score. The scale demonstrated good internal consistency, with a Cronbach’s alpha of 0.85.

#### Mother-adolescent communication

At the study visit, youth completed the Parent-Adolescent Communication Scale [43] was administered by a study interviewer, with youth providing reflections on their communication with their mother. The Scale includes 10 statements rated on a 4-point Likert scale (e.g., “My mother and I can talk about almost anything,” “My mother sometimes does not listen to me”; Strongly disagree, Disagree, Agree, Strongly Agree). Summary scores were computed as an average score with a range from 1 to 4, with 4 reflecting the best possible communication. The scale demonstrated good internal consistency, with a Cronbach’s alpha of 0.83.

#### Youth fruit and vegetable intake

Youth independently completed the Block Kids Food Screener (BKFS). This screener presents 39 food and beverage categories and asks youth to report how many times they consumed them in the last week [44]. For any foods or beverages they consumed, they were asked to indicate how much they consumed each time. Quantity estimates were presented in a straightforward way; for example, youth were asked how many bowls, glasses, bottles, etc. they usually consumed. Responses were analyzed by the publisher, Nutritionrequest [44]. The BKFS has been independently validated against 24-hour dietary recalls. The primary BKFS variables used in the present analysis are estimated intake in cup equivalents per day of fruit and vegetables, separately, as calculated by the publisher [44]. Vegetable estimates exclude potatoes and legumes. Fruit estimates include real fruit juice, consistent with current public health guidelines [45]. Responses were recorded as a cup serving size per day.

#### Youth fast food intake

Mothers were asked to report the number of times in the last week that their child had eaten food from “a fast food restaurant like McDonald’s, Burger King, KFC, or Taco Bell.” Responses were recorded as less than once a week, 1–2 times per week, or 3 + times per week.

#### Youth physical activity and screen time

Youth completed the Block Physical Activity Screener (BPAS) independently. This screener presents 9 categories of leisure and school activities, chores and part time jobs [46]. For each, it asks the number of times the youth participated in this activity in the past week, and for how long. The primary BPAS physical activity variables used in this analysis are estimated moderate activity minutes per day and vigorous activity minutes per day, all continuous variables. The screen time variable used in this study was also drawn from the BPAS and reflected the number of hours per day spent on TV or computer use, including watching television, playing video games, and browsing the internet. Responses were analyzed by the publisher, Nutritionrequest [44].

#### Youth sleep

Adolescents answered questions independently about their typical bedtime on school nights and weekend/vacation nights, as well as their typical wake times on school days and weekend/vacation days. From these, typical sleep duration was calculated separately for school nights and non-school nights, and a weighted average of these values (five school nights/two non-school nights) was calculated to reflect the adolescents’ average hours of sleep per night. 

#### Weight status

Height and weight of the adolescent were measured. Barefoot standing height was measured to the nearest 0.1 cm using a wall-mounted stadiometer (Seca 222; Seca North America). Weight was measured to the nearest 0.1 kg using a bioimpedance scale (Tanita TBF-300 A Body Composition Analyzer; Tanita Corporation of America). BMI percentile scores were calculated per Centers for Disease Control [47]. BMI percentiles reflect how the adolescent’s BMI compares to adolescent of their same age and sex in a normative sample.

#### Other covariates

At their first CHAMACOS visit, mothers reported their highest level of school attended or completed; responses were categorized as < = sixth grade education, 7th − 12th grade without high school graduation, and high school graduate or higher. At the 14y visit, mothers reported their usual monthly household income and the number of people it supported. These values were compared to Census Bureau 2013 thresholds for poverty level, and a poverty level was assigned categorically as follows: below the federal poverty level, 100–200% of the federal poverty level (i.e., low income but not in poverty), and > 200% of the poverty level. Mothers’ weight status was assessed by calculating BMI from their height and weight measured at the adolescent’s age 14 visit (same methods as for adolescent).

### Statistical analysis

IBM SPSS Statistics 28 was used for descriptive statistics and Mplus for structural equation modeling (SEM) analysis to test overall model fit and hypothesized associations [48]. Missing data were computed under maximum likelihood estimation. To assess the construct validity of the measurement models for latent constructs, a confirmatory factor analysis (CFA) was conducted separately for the items of familism, fruit and vegetable intake and physical activity. After ensuring the adequate fit for the measurement models, the SEM path analysis was conducted, separately for maternal monitoring and for mother-adolescent communication. Maternal education level, family poverty level and mother’s weight status were added as covariates. To account for potential correlations and mutual influences among parenting and behavioral variables, we utilized a regression-based structural modeling approach [49]. This approach assumes no unmeasured confounding of the variables to ensure the various path coefficients are not biased. After testing the total sample, a multi-group analysis was conducted to compare outcomes between boys and girls groups. Model fit was evaluated using the combination of the comparative fit index (CFI), Tucker-Lewis Indes (TLI) and the root-mean-square error of approximation (RMSEA). Criteria for an acceptable model fit included CFI and TLI values of 0.90 or higher, with RMSEA values of 0.08 or less indicating adequate fit and 0.05 or less indicating close fit [50, 51].

## Results

### Descriptive statistics

Table 1 presents the demographic information for the sample. On average, mothers were 40.6 years old (SD = 5.3), and the majority had low levels of education, with only 22.3% having completed high school or higher. Most mothers were married (76.7%) and born in Mexico (87.2%). Nearly half of the adolescents were boys (47.2%), while 52.8% were girls. A large proportion of families (70.4%) were living at or below the poverty line. Overall, 45.8% of adolescents were non-overweight (normal or underweight), 19.3% were overweight, and 33.1% were obese. Table 2 shows descriptive statistics of the study variables. Independent samples t-tests showed that girls reported significantly higher maternal monitoring and screen time compared to boys. Boys reported significantly higher levels of fruit and vegetable intake, moderate and vigorous physical activity than girls. No significant gender differences were found for other variables. The skewness and kurtosis of all variables were checked to ensure normality. A square root transformation was applied to vegetable consumption to attain normal distribution (skewness 2.02 to 0.84, Kurtosis 5.64 to 0.96) [52]. Table 3 presents the bivariate correlations among the study variables. The three familism indicators were moderately correlated with one another, and fruit and vegetable intake also showed a moderate correlation.

**Table 1:** Characteristics of study participants

Variable	No.	%
Mother’s age (M ± SD)		40.6 ± 5.3
Maternal education level		
<= 6th grade	187	42.6%
7-12th grade	154	35.1%
>= High school graduate	98	22.3%
Maternal Marital Status		
Married	337	76.7%
Separated	43	9.8%
Divorced	14	3.2%
Widowed	5	1.1%
Single	40	9.1%
Mother’s country of birth		
US	49	11.2%
Mexico	383	87.2%
Other	5	1.1%
Adolescent self-reported gender		
Boys	207	47.2%
Girls	232	52.8%
Household poverty category		
At or below poverty	309	70.4%
Poverty − 200%	127	28.9%
> 200% Poverty	2	0.5%

**Table 2:** Descriptive statistics

	Scale	Total Sample	Boys	Girls
		Mean ± SD	Mean ± SD	Mean ± SD
Familism - Obligation	1–5	3.87 ± 0.55	3.89 ± 0.51	3.86 ± 0.51
Familism - Referent	1–5	4.17 ± 0.46	4.14 ± 3.51	4.19 ± 0.44*
Familism - Support	1–5	4.06 ± 0.41	4.06 ± 9.51	4.06 ± 0.37
Mother-Adolescent Communication	1–4	2.70 ± 0.46	2.71 ± 2.51	2.70 ± 0.40
Maternal Monitoring	1–5	4.04 ± 0.66	3.91 ± 3.51	4.15 ± 0.66*
Fruit Intake (cups/day)	1–7	1.46 ± 1.16	1.57 ± 0.51*	1.37 ± 1.22
Vegetable Intake (cups/day)	1–7	0.75 ± 0.61	0.88 ± 0.67*	0.64 ± 0.53
Fast Food Intake (less than once a week, 1–2 times per week, 3 + times per week)	1–3	0.81 ± 0.53	0.82 ± 0.54	0.79 ± 0.53
Moderate Physical Activity (minutes/day)	-	73.26 ± 57.80	82.34 ± 64.01*	65.57 ± 50.85
Vigorous Physical Activity (minutes/day)	-	49.72 ± 51.89	62.52 ± 59.99*	38.88 ± 40.96
Screen Time (hours/day)	1–6	4.33 ± 1.35	4.23 ± 0.51	4.41 ± 1.31*
Sleep (hours/day)	-	8.99 ± 1.02	8.99 ± 3.51	8.98 ± 1.07
BMI percentile	0–100	74.64 ± 27.71	73.97 ± 28.64	75.23 ± 26.93

*SD*  Standard deviation

*p < .05 for gender difference based on independent samples

### Measurement model

As shown in Table 3, the factor loading for latent variables from the CFA indicated that observed variables loaded significantly onto their respective latent variables of familism, fruit and vegetable intake and physical activity. These robust factor loadings demonstrated that the indicators coherently represented the underlying cultural or behavioral dimensions. By utilizing these latent constructs rather than single-item measures, this approach ensures that the estimated associations reflect relationships between well-defined, multi-dimensional constructs while accounting for measurement error. Furthermore, all measurement models demonstrated satisfactory fit indices, confirming the structural validity of the framework.

### Path analysis for the total sample

#### Mother-adolescent communication

**Fig. 2:**
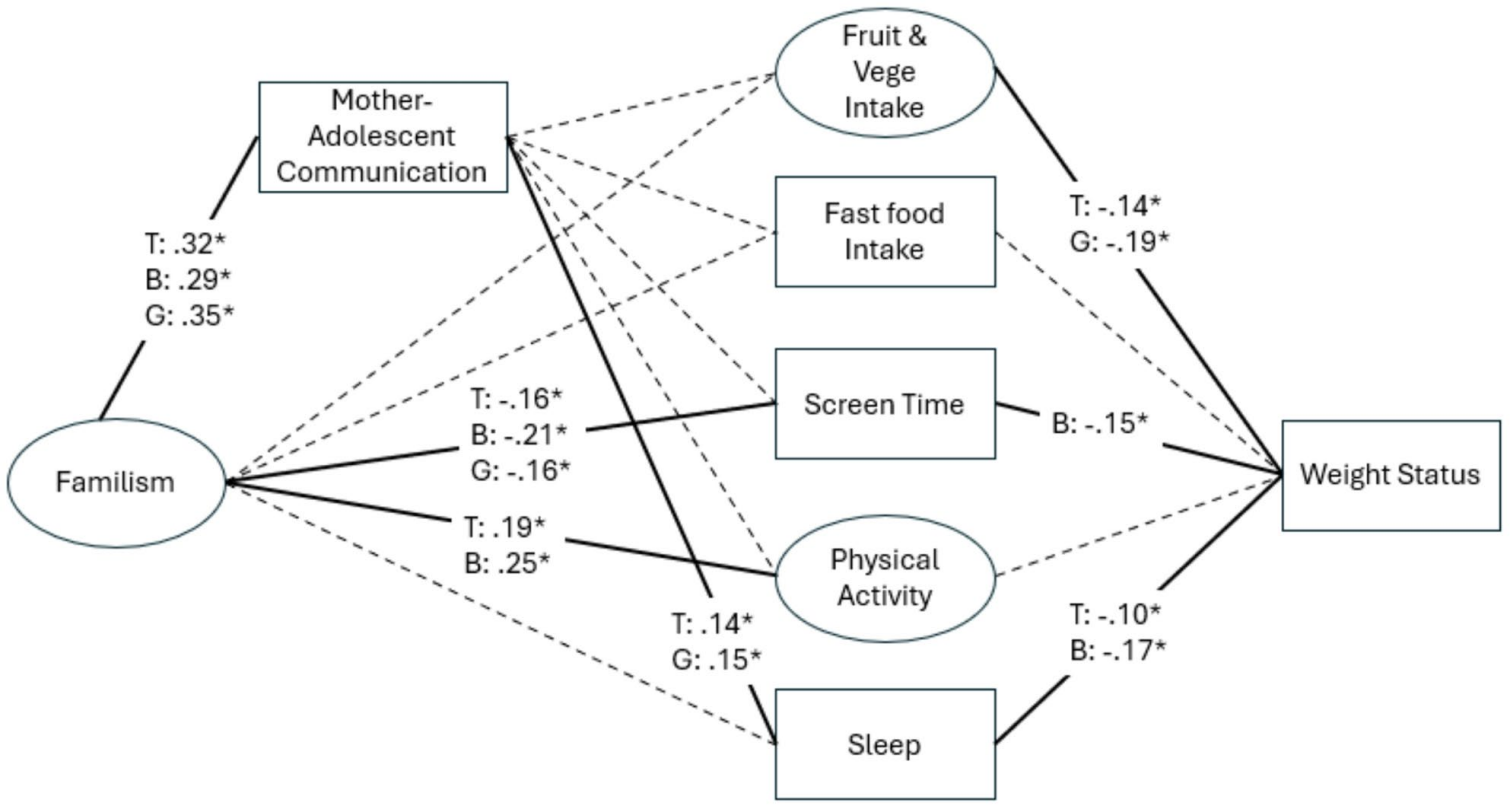
Final model of mother-adolescent communication with standardized coefficients. Although not shown in the figure, maternal education level, household poverty level and maternal weight status were included as covariates. Ellipses and rectangles represent unobserved latent and observed variables, respectively. Dashed lines indicate a nonsignificant path. Lines indicate associations, not causal pathways. *indicates a statistically significant path (*p* < .05) and is noted by a solid line. T: Total sample. B: Boys. G: girls

Model fit was satisfactory (CFI = 0.92, TLI = 0.87, RMSEA = 0.05). All paths are detailed in Table 4 and significant paths are presented in Fig. 2. Familism was positively related to mother-adolescent communication. Familism was positively associated with physical activity and negatively with screen time. Mother-adolescent communication was positively related to sleep duration. Fruit & vegetable consumption and sleep were negatively related to weight status.

#### Maternal monitoring

**Fig. 3:**
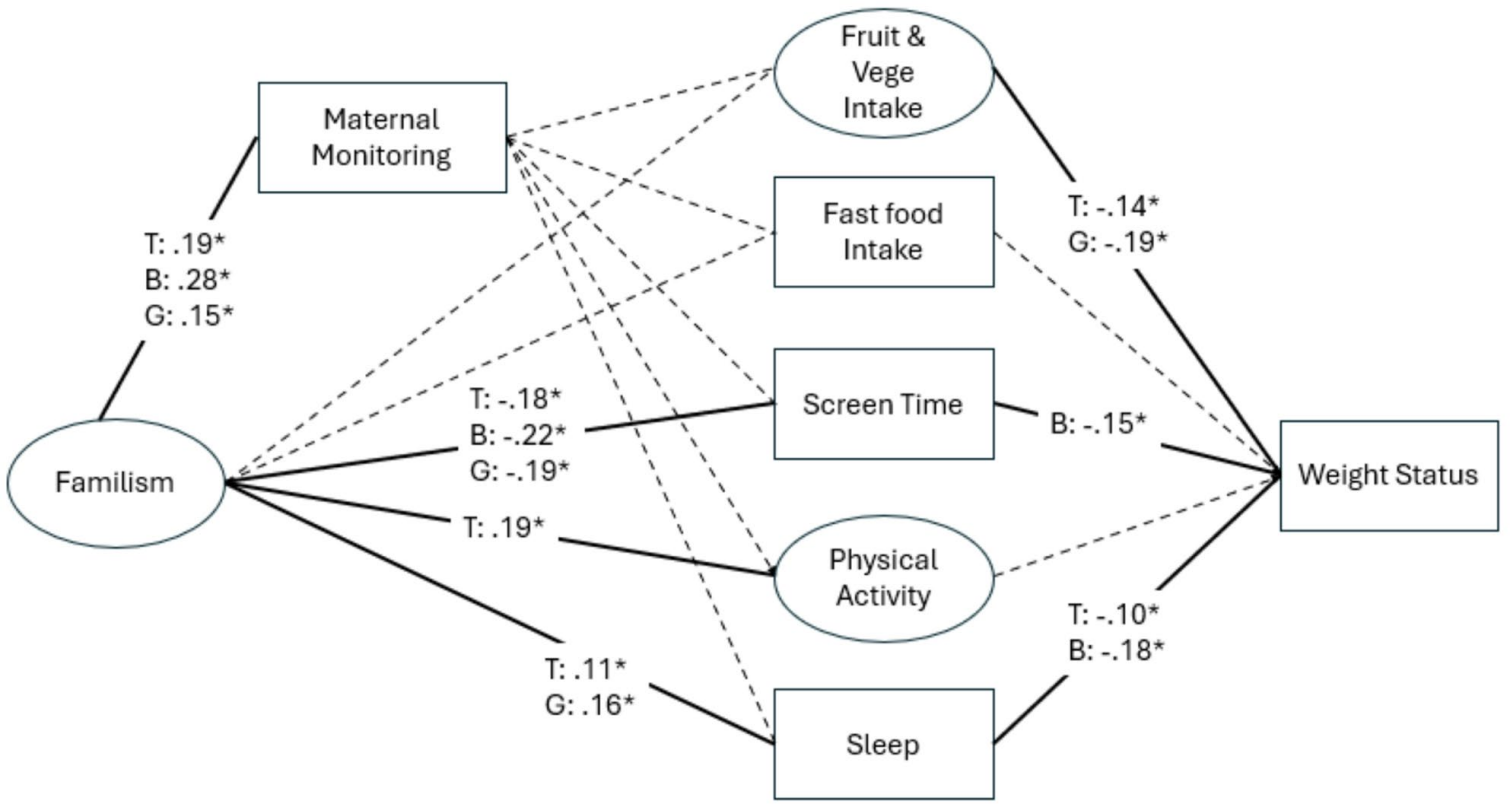
Final model of maternal monitoring with standardized coefficients. Although not shown in the figure, maternal education level, household poverty level and maternal weight status were included as covariates. Ellipses and rectangles represent unobserved latent and observed variables, respectively. Dashed lines indicate a nonsignificant path. Lines indicate associations, not causal pathways. *indicates a statistically significant path (*p* < .05) and is noted by a solid line. T: Total sample. B: Boys. G: girls

Model fit was satisfactory (CFI = 0.92, TLI = 0.86, RMSEA = 0.05). All paths are detailed in Table 5 and significant paths are presented in Fig. 3. Familism was positively related to maternal monitoring. Regarding the significant associations to weight-related behaviors, familism was positively associated with physical activity and sleep and negatively with screen time. Maternal monitoring was not related to any weight-related behaviors. Fruit and vegetable intake and sleep were negatively related to weight status.

### Multi-group analysis for boys and girls

#### Mother-adolescent communication

Model fit was satisfactory (CFI = 0.95, TLI = 0.91, RMSEA: 0.04) for the multi-group.

SEM. Significant paths for boys and girls are shown in Fig. 2. Both boys’ and girls’ familism were positively related to parent-adolescent communication. Regarding the significant associations to weight-related behaviors, boys’ and girls’ familism were both inversely related to screen time. Boys’ familism but not girls’ was positively related to physical activity. Girls’ communication with their mothers was positively associated with girls’ sleep duration. Girls’ fruit and vegetable intake and boys’ screen time and sleep duration were negatively related to their weight status.

#### Maternal monitoring

Model fit was satisfactory (CFI = 0.94, TLI = 0.90, RMSEA = 0.04). Significant paths are detailed in Fig. 3. Boys’ and girls’ familism were positively related to maternal monitoring. Regarding the significant associations to weight-related behaviors, both boys’ and girls’ familism were inversely related to screen time and girls’ familism was positively related to sleep duration. Girls’ fruit and vegetable intake and boys’ screen time and sleep duration were negatively related to their weight status.

**Table 3:** Correlation and Confirmatory Factor Analysis (CFA)

		1	2	3	4	5	6	7	8	9	10	11	12
1	Familism - Obligation	1											
2	Familism - Referent	.60^*^	1										
3	Familism - Support	.61^*^	.58^*^	1									
4	Mother-Adolescent Communication	.23^*^	.22^*^	.30^*^	1								
5	Maternal Monitoring	.13^*^	.13^*^	.21^*^	.43^*^	1							
6	Fruit Intake	.06	.04	.06	− .03	− .01	1						
7	Vegetable Intake	.06	.02	.06	− .03	− .01	.58^*^	1					
8	Fast Food Intake	− .04	− .02	− .07	.09	.01	− .06	− .13^*^	1				
9	Screen Time	− .11^*^	− .18^*^	− .12^*^	− .11^*^	− .05	− .02	− .06	.03	1			
10	Moderate Physical Activity	.10^*^	.11^*^	.11^*^	.04	− .07	.24^*^	.22^*^	.02	− .07	1		
11	Vigorous Physical Activity	.09	.12^*^	.05	.03	− .01	.25^*^	.27^*^	.03	− .11^*^	.43^*^	1	
12	Sleep	.07	.12^*^	.08	.15^*^	.06	.07	.02	− .05	− .27^*^	.07	− .05	1
13	Weight Status	.04	.07	.02	.06	− .04	− .14*	− .09	− .02	− .05	− .04	− .01	− .06
CFA	Familism	.78*	.75*	.78*									
	Fruit and Vegetable Intake						.79*	.72*					
	Physical Activity										.77*	.56*	

*p < .05

**Table 4:** Standardized path coefficients for the mother-adolescent communication model (Total Sample and by Gender)

Paths	Total			Boys			Girls		
	Beta	SE	*p*	Beta	SE	*p*	Beta	SE	*p*
Familism - Mother-Adolescent Communication	.32*	**0.05**	**0.00**	.29*	**0.80**	**0.00**	.35*	**0.07**	**0.00**
Familism - Fruit & Vege Intake	.11	0.07	0.10	.18	0.10	0.09	.05	0.08	0.55
Familism - Fast-food Intake	− .03	0.06	0.65	.02	0.08	0.85	− .05	0.08	0.49
Familism - Screen Time	− .16*	**0.06**	**0.01**	− .21*	**0.08**	**0.01**	− .16*	**0.08**	**0.04**
Familism - Physical Activity	.19*	**0.08**	**0.02**	.25*	**0.11**	**0.02**	.16	0.11	0.14
Familism - Sleep	.08	0.06	0.18	.05	0.08	0.85	.11	0.08	0.16
Mother-Adolescent Communication - Fruit & Vege Intake	− .09	0.06	0.15	− .06	0.08	0.44	− .09	0.08	0.23
Mother-Adolescent Communication - Fast-food Intake	− .09	0.05	0.09	− .09	0.07	0.25	− .09	0.07	0.22
Mother-Adolescent Communication - Screen Time	− .07	0.05	0.19	− .03	0.08	0.65	− .07	0.07	0.31
Mother-Adolescent Communication -Physical Activity	− .02	0.07	0.76	.02	0.10	0.87	− .04	0.10	0.67
Mother-Adolescent Communication - Sleep	.14*	**0.05**	**0.01**	.12	0.08	0.12	.15*	**0.07**	**0.03**
Fruit & Vege Intake - Weight Status	− .14*	**0.06**	**0.02**	− .06	0.09	0.46	− .19*	**0.08**	**0.02**
Fast food Intake - Weight Status	− .04	0.05	0.35	− .06	0.07	0.34	− .03	0.06	0.62
Screen Time - Weight Status	− .09	0.05	0.06	− .15*	**0.07**	**0.04**	− .04	0.07	0.60
Physical Activity - Weight Status	.02	0.06	0.08	− .06	0.09	0.51	.01	0.09	0.88
Sleep - Weight Status	− .10*	**0.05**	**0.04**	− .17*	**0.07**	**0.02**	− .04	0.07	0.54

Coefficients are standardized. Solid paths in corresponding Fig. 2, which are boldfaced in this table, indicate statistically significant associations (*p < .05)

**Table 5:** Standardized path coefficients for the maternal monitoring model (Total Sample and by Gender)

Paths	Total			Boys			Girls		
	Beta	SE	*p*	Beta	SE	*p*	Beta	SE	*p*
Familism - Maternal Monitoring	.19*	**0.05**	**0.00**	.29*	**0.08**	**0.00**	.15*	**0.07**	**0.03**
Familism - Fruit & Vege Intake	.09	0.06	0.15	.17	0.10	0.09	.01	0.08	0.88
Familism - Fast-food Intake	− .06	0.05	0.28	− .02	0.08	0.83	− .09	0.07	0.24
Familism - Screen Time	− .18*	0.05	**0.00**	− .22*	**0.08**	**0.01**	− .19*	**0.07**	**0.01**
Familism - Physical Activity	.19*	**0.08**	**0.03**	.26*	**0.11**	**0.01**	.15	0.10	0.14
Familism - Sleep	.11*	**0.06**	**0.04**	.06	0.08	0.50	.16*	**0.07**	**0.01**
Maternal Monitoring - Fruit & Vege Intake	− .05	0.06	0.41	− .06	0.08	0.46	− .19*	**0.08**	**0.02**
Maternal Monitoring - Fast-food Intake	.03	0.05	0.49	− .02	0.08	0.83	.03	0.07	0.67
Maternal Monitoring - Screen Time	− .00	0.05	0.96	− .02	0.08	0.81	.00	0.07	0.96
Maternal Monitoring - Physical Activity	− .12*	**0.06**	**0.05**	− .05	0.10	0.63	− .03	0.10	0.76
Maternal Monitoring - Sleep	.05	0.05	0.37	.08	0.08	0.26	.01	0.07	0.94
Fruit & Vege Intake - Weight Status	− .14*	**0.06**	**0.02**	− .06	0.08	0.47	− .19*	**0.08**	**0.02**
Fast food Intake - Weight Status	− .04	0.05	0.36	− .07*	**0.07**	**0.02**	− 003	0.06	0.62
Screen Time - Weight Status	− .00	0.05	0.96	− .15*	**0.07**	**0.03**	− .03	0.07	0.61
Physical Activity - Weight Status	− .01	0.06	0.82	− .07	0.09	0.48	.02	0.10	0.85
Sleep - Weight Status	− .10*	**0.05**	**0.04**	− .18*	**0.07**	**0.02**	− .03	0.07	0.56

Coefficients are standardized. Solid paths in corresponding Fig. 3 , which are boldfaced in this table, indicate statistically significant associations (*p < .05)

## Discussion

The current study examined associations among familism, maternal parenting processes, and weight-related behaviors among Mexican-origin adolescents in an agricultural area of California. Given the cross-sectional nature of the data, the analyses were intended to evaluate whether the observed patterns are consistent with the proposed theoretical model rather than to establish causal or temporal ordering [53, 54]. Accordingly, the findings should be interpreted as foundational evidence that informs future hypothesis-driven research, providing an empirically grounded framework for longitudinal studies to formally examine directional pathways over time.

We found adolescent familism was positively associated with both mother-adolescent communication and maternal monitoring. While mother-adolescent communication was positively linked to adolescent sleep duration, maternal monitoring was not significantly related to any of the weight-related behaviors. Also, we found familism was a health-promoting correlate, showing a positive association with physical activity and sleep and a negative association with screen time. Among the weight-related behaviors examined simultaneously, fruit and vegetable intake and sleep were negatively associated with weight status. Gender differences were also found; girls’ familism was positively associated with their sleep, whereas boys’ familism was positively related to their physical activity. For girls, fruit and vegetable intake was negatively associated with their weight status, whereas for boys, screen time was negatively associated with weight status.

The consistent positive associations between familism and parenting processes aligns with Ecological Systems Theory, which posits that macrosystem cultural values, such as familism, can shape interactions within the microsystem [37, 38], such as mother-adolescent communication and maternal monitoring [55]. Familism values are thought to foster prosocial behaviors and traits as Latino cultural patterns often emphasize positivity and downplay negativity in relational contexts [16, 56]. These findings support conceptual frameworks suggesting that familism contributes to cohesion and supportive family relationships, while reducing negativity and conflictual dynamics within families [56–58]. Adolescents who strongly endorse familism tend to maintain close, respectful relationships with their parents, which may encourage open communication and greater disclosure about their activities [59]. Thus, supportive communication and monitoring practices are products of both the immediate family system and the Latino cultural emphasis on family unity.

The current study identifies familism as a correlate of several weight-related behaviors, as found by its positive associations with physical activity and sleep, and its negative association with screen time. Prior research has documented robust associations between familism and health, highlighting both direct links and stress-buffering patterns, whereby higher levels of familism are associated with greater perceived social support and lower stress-related health risks [60, 61]. The positive association between familism and physical activity aligns with the previous finding that adolescents who reported a stronger connection with their family also engaged more frequently in physical activity than adolescents who reported lower familism [62]. Similarly, the inverse association between familism and screen time is consistent with previous findings suggesting that greater screen use is associated with emotional distress among adolescents [63]. In this context, higher familism may be linked to lower screen time through its association with emotional support, family engagement, and structured family routines, although the direction of these associations cannot be determined in cross-sectional data.

However, familism was not significantly associated with adolescents’ food consumption. This pattern is consistent with prior studies indicating that familism was not directly related to youth eating behaviors but was instead associated with dietary outcomes through broader family processes, such as family functioning or family mealtime interactions [64, 65].

When all five weight-related behaviors were examined simultaneously, only one significant association between family factors and adolescent weight status emerged. Specifically, maternal communication was positively associated with adolescent sleep. Open parent-child communication, defined as the reciprocal exchange of both factual and emotional information, has been consistently associated with healthier family dynamics and greater emotional security among adolescents [60, 61]. Healthy sleep patterns, such as sufficient duration and good quality, are more likely to emerge in environments that feel emotionally secure and supportive, where adolescents experience both psychological and physiological safety [66]. Adolescents who experience consistent and open communication with their mothers may feel less stressed, more emotionally regulated, and psychologically safe, which are conditions conducive to falling and staying asleep [67]. In addition, open communication was associated with longer sleep duration, a pattern consistent with theories suggesting that emotionally supportive environments may co-occur with healthier sleep through reducing pre-sleep rumination and improving sleep onset [68]. Spending time with family may be particularly important for Mexican American adolescents, as traditional Latino cultural values emphasize family connectedness and shared daily routines [69]. For instance, Mexican adolescents who spent more time with their families tended to sleep longer [29]. Taken together, these findings suggest that warm, communicative, and engaged family environments are associated with healthier sleep behaviors among adolescents.

Interestingly, we did not find significant associations between maternal general monitoring and adolescent weight-related behaviors in our sample. Much of the existing literature on parental monitoring has focused on its role in reducing adolescent health-risk behaviors, such as smoking, alcohol use, and sexual activity, rather than everyday lifestyle behaviors like diet, physical activity, or screen time [70, 71]. The only study we are aware of that examined parental monitoring in relation to weight-related behaviors among Latino adolescents found that higher levels of monitoring were significantly associated with healthier behaviors and lower weight status [26]. This difference may reflect differences in study context and measurement. For instance, the prior study focused on an urban Latino population and incorporated adolescent reports of both maternal and paternal monitoring, potentially capturing a broader view of parental involvement. In contrast, our study examined rural Mexican-origin families and relied solely on adolescents’ perceptions of maternal monitoring. These differences in cultural context, family structure, or the scope of monitoring captured may be related to the observed associations; however, further research is needed to directly test these possibilities.

Among the weight-related behaviors examined, fruit and vegetable intake and sleep duration were negatively associated with weight status. Fruits and vegetables are nutrient-dense, low-calorie foods that are high in water and dietary fiber, characteristics that have been associated with greater satiety and lower overall caloric intake in prior research and may help contextualize the observed associations [72, 73]. Similarly, longer sleep duration has been linked to lower weight status among adolescents [74]. Adequate sleep plays a critical role in regulating appetite-related hormones, such as ghrelin, which stimulates hunger, and leptin, which signals satiety [75]. Sleep deprivation disrupts this hormonal balance, often resulting in increased appetite and energy intake [75, 76]. Furthermore, adolescents who sleep fewer hours are awake for longer periods, which increases opportunities for late-night eating and snacking on energy-dense foods [77].

Gender-specific patterns emerged in how maternal parenting practices and familism were associated with weight-related behaviors among Latino adolescents. For boys, familism was positively related to higher physical activity while we did not find such connection among girls. Research suggests that Latino boys are often socialized to learn active, outgoing and provider roles within the family context [78]. Familism was associated with higher physical activity among boys, a pattern consistent with gendered norms identified in prior research [79]. In contrast, the significant positive association between mother-daughter communication and girls’ sleep duration suggests that girls may rely more heavily on relational and emotional aspects of family dynamics. But this is only a speculation as gender and/or sex differences were typically controlled for among Latino adolescents in literature [29].

Furthermore, we observed a negative association between screen time and weight status. It was a counterintuitive finding, as previous literature generally links greater screen time to higher weight status [80]. However, similar patterns have been reported in studies of younger children. For instance, first-generation Mexican American children aged 6–11 had higher obesity prevalence despite engaging in less screen time than their more acculturated (third generation) peers [81]. This may suggest that screen time is associated with weight outcomes independently of family constructs.

One possible explanation lies in the contextual nature of screen time behaviors in rural, low-income Mexican-origin families. In this particular context, screen use may not reflect passive or sedentary leisure, but rather a limited activity often shared among multiple family members. Additionally, traditional gender roles and family labor expectations could contribute to regulating screen time; adolescent boys in immigrant families may have chores or work responsibilities that limit their leisure screen hours. It is also possible that the current measurement of screen time, which focused on watching and using a computer to play games and browse the internet, may have contributed to the pattern of findings observed, as unmeasured forms of screen may operate differently than the types of screen activities assessed in this study.

### Limitations

This study draws on rich data from the CHAMACOS cohort, which offers valuable insights into the development of the Latino adolescents primarily of immigrant Mexican families living in a low-income agricultural community. However, several limitations should be noted. First, the generalizability of the study is limited by the sample’s geographic and socioeconomic specificity, as well as the exclusive focus on mothers and a lack of representation of gender-diverse, nonconforming, or gender-expansive adolescents. Additionally, many of the measures relied on self-reported measures, which are subject to recall bias and social desirability effects. Sleep duration, for example, was derived exclusively from participants’ reported bedtimes and wake times, excluding the time spent falling asleep and any awakenings during the night. Furthermore, the simultaneous assessment of multiple parenting and behavioral variables may be subject to residual bias [82], as shared method variance and correlated reporting tendencies may inflate or obscure associations among constructs measured at the same time point. Finally, potential cultural variation in the interpretation of survey items may affect the robustness and interpretability of some findings.

## Conclusions

The findings from this study underscore the importance of integrating cultural values and family functioning into the understanding of adolescent weight-related behaviors and weight status. In particular, the role of familism and maternal parenting processes should be considered in developing culturally responsive strategies to support healthy weight-related behaviors, such as healthy food intake and adequate sleep, among Mexican American adolescents. The observed gender differences and cultural-contextual variations point to the need for culturally sensitive and gender-responsive approaches in adolescent weight status research and interventions. Future research should distinguish different dimensions of screen use, including content (e.g., active gaming, sports-related media, educational use), platform (e.g., television, smartphones, gaming consoles), and social context (e.g., solitary vs. family-based use). Additionally, longitudinal designs and mixed-method approaches would provide a deeper understanding of how cultural values and maternal parenting processes contribute to adolescent obesity over time, particularly within underserved immigrant communities.

## Data Availability

The datasets generated during and/or analyzed during the current study are not publicly available due to protection of personal identifiers and the sensitive nature of these data, but are available from the corresponding author on reasonable request.

## Abbreviations

BKFS: Block Kids Food Screener
SEM: Structural Equation Modeling
CFA: Confirmatory Factor Analysis
